# Experimental Laboratory Testing on Behavior of Dowels in Concrete Pavements

**DOI:** 10.3390/ma13102343

**Published:** 2020-05-20

**Authors:** Andrea Zuzulova, Jiri Grosek, Michal Janku

**Affiliations:** 1 Department of Transportation Engineering, Faculty of Civil Engineering, Slovak University of Technology in Bratislava, 810 05 Bratislava, Slovakia; andrea.zuzulova@stuba.sk; 2 Transport Infrastructure Department, Transport Research Centre−CDV, 636 00 Brno, Czech Republic; michal.janku@cdv.cz

**Keywords:** dowel, concrete pavement, laboratory testing, concrete beams

## Abstract

This paper describes the testing of effectiveness and behavior of dowels placed in transversal joints of concrete pavements, while focusing on dimensions and quality of commonly used materials. The analysis uses experimental tests in laboratory conditions which were performed independently in the Czech Republic and Slovak Republic. The comparison of quality as well as potential use of alternative materials of dowels is made with the use of developed tests focusing on main requirements, such as longitudinal displacement in cement concrete, resistance of coating to damage, and reduced potential to concrete damage. Furthermore, the paper describes and compares loading results of the relative concrete deformations around dowels by strain gauges that were analyzed. Results of deformations on beams with an inserted dowel and the findings that were observed during the measurement are presented.

## 1. Introduction

This article is an outcome of research in the Czech and Slovak Republics that aimed perform measurements of stress on beams with an inserted dowel, to assess their effectiveness and to test the concentration of tensile stress in the concrete around dowels intended for jointed plain concrete pavements (JPCP).

Together with the aggregate interlock, dowels are a solution to improve interaction between slabs at transversal joints (LTE—load transfer efficiency) of concrete pavements. However, this technological complication may not have a long-term effect in cases when concrete is damaged along the dowels and their function may be lost. An analysis of the current JPCP loading showed another potential complication in the form of existence of high concentrations of tensile stress of concrete at faces of transversal joints around dowels and potential occurrence of microcracks in this area. Theoretical modelling results by the finite element method (FEM) method show that stress in the concrete slab increases under static and thermal loading, particularly in the vicinity of dowels [1]. The FEM calculation analysis was used for the JPCP design for many cases [2,3]. First of all, 2D FEM ingress was applied in concrete pavement modelling [4,5,6]. More accurate input data and results which cannot be achieved in 2D modelling start to appear with the development of computer technology and new 3D software tools [7,8,9,10]. 

Many models deal with the dowel−concrete interaction through theoretical modelling and applied nonlinear calculation along the whole length of the dowel based on an analysis of stress distribution [8,11,12,13]. Although FEM models focused on dowels of JPCPs, only several of them dealt with stresses and practical verification of damage behavior around dowels by laboratory testing [14,15]. 

The issue of the real behavior of dowels in transversal joints of JPCPs and its long-term monitoring is described by several foreign authors [3,16,17]. An important study summarizing the behavior of JPCPs with dowels is presented in [18]. The authors point out that many of the epoxy-coated dowels retrieved from in-service pavements revealed that the epoxy coating was debonded from the steel dowel and the surface of the steel dowel under the coating was pitted and rusted. A review of plastic-coated dowels in the USA indicated that those dowels were in excellent condition after 40 years. Based on the coring of older pavement projects, fibre-reinforced polymer (FRP) with polyester resin and E-glass, stainless steel-clad dowels and concrete filled stainless steel tubes or pipes were studied as an alternative solution. Another issue that needs to be monitored is the quality of the commonly used dowel coating marked polyethylene (PE) plastic, which is soft and is often pressed into concrete, thus reducing the dowel function—LTE. Last but not least, the concerning issue is the correct position of dowels, whose significant deflection may lead to cement concrete road pavement damage. Another important issue is the development of alternative materials.

Although the real behavior of dowels by laboratory testing, which verifies results of FEM modelling, dowels effectiveness, behavior during loading and alternative dowel materials, was not satisfactory, it was studied in detail. The cooperation of Transport Research Centre and the Faculty of Civil Engineering, STU Bratislava, was based on analyses of dowel effectiveness, since the effect of dowel dimensions on cement concrete slabs behavior is still not completely known and clear in the field of road pavements design. In its first phase, the experimental research in the Czech Republic and Slovakia focused on overall durability, deformation properties, and the damage type of beams with inserted dowels. 

Furthermore, cooperation was established with the aim of focused research and the development of alternative materials suitable for installations in motorway and highway structures as well as airport pavements. Nowadays, a single dowel type is used in the Czech Republic (length 500 mm, diameter 25 mm, PE plastic coating thickness 0.3 mm); in Slovakia and foreign countries (apart from Germany and Austria) dowel dimensions are prescribed based on the concrete pavement thickness [19]. In addition, it is necessary to deal with the issue of reinforcement elements in cement concrete slabs in transversal direction and the issue of coating type, where coating from more durable plastic material and fiberglass was developed as an alternative to the existing one.

The material parameters of common dowels comply with Czech standard ČSN 736123–1 and European standards EN 13877–1 and EN 13877–3 [20,21,22]. The standards describe the dowel as made of plain steel, coated and installed in joints of adjacent concrete pavement slabs to improve load transfer effectiveness to prevent the slabs from vertical displacement and to comply with the requirements of EN 13877–3 regarding material quality. The Czech standard ČSN 73 6123-1 establishes the following requirements for the position of dowels measured after compaction, from which they may not deviate regarding the position specified in the project documentation:

- tilt of the dowels relative to the length of 500 mm (the horizontal and vertical difference of dowel ends) may be up to 25 mm, but this must be met by a minimum of 75% of dowels in the joint and the remaining 25% of dowels in the joint may be up to 40 mm;
- tolerance in vertical translation (depth under the pavement surface) may be up to 30 mm, but this value must be met by a minimum of 75% of dowels in the joint and the remaining 25% of dowels in the joint may be up to 50 mm;
- tolerance in longitudinal translation (divergence towards transversal joint) may be up to 75 mm, but this value must be met by a minimum of 75% of the dowels in the joint and the remaining 25% of dowels in the joint may be up to 120 mm.

Durability of dowels must be ensured by protective factory-applied coating or applied in a construction site in compliance with national standards applicable in the country of use. Anticorrosion measures must meet the requirements of national standards or regulations in the country of use. The standards define dowels used for class CB I cement concrete road pavements, which must be of the minimum diameter of 25 mm and minimum length of 500 mm. The whole length of the dowel must be covered with a thin plastic film of 0.3 mm thickness, which must guarantee corrosion protection and at the same time allow the dowel slip in concrete. The coating must meet special guideline (TP 136) requirements. Different coatings can be used for class CB II. The coating for CB I must be factory-applied. However, the manufacturer must document thickness measurement of the dowel plastic coating as well as the dowel tensile strength R_m_ (MPa), in the range of 1 to 2 500 dowels. The requirement of steel quality to achieve class S 235, which is commonly used, and the quality of coating is not monitored.

Dowels must have tensile strength Rm at least 250 MPa in compliance with EN ISO 15630–1 [23]. Diameter and diameter tolerance of dowels must comply with the requirements of EN 10060. The length tolerance must be maximum ±10 mm. Dowels must by straight, free of sharp projections and other unevenness, and must have smooth ends free of sharp projections that would project over the dowel diameter [24].

The above mentioned sources and information make it obvious that the issue of using dowels in JPCPs may significantly affect their durability. Some of the main aims of the study include verifying behavior of different types of dowels (diameters, quality of used materials, and their position/location towards the transversal joint) in laboratory conditions and show shortcomings when used. Another aim of the research activities is to formulate research conclusions and results while taking into account foreign authors’ experience, which would subsequently be integrated in effective regulations and use in practice in Slovakia and the Czech Republic.

## 2. Materials and Methods

### 2.1. Czech Loading Test

#### 2.1.1. Cement Concrete Parameters 

**Figure 1:**
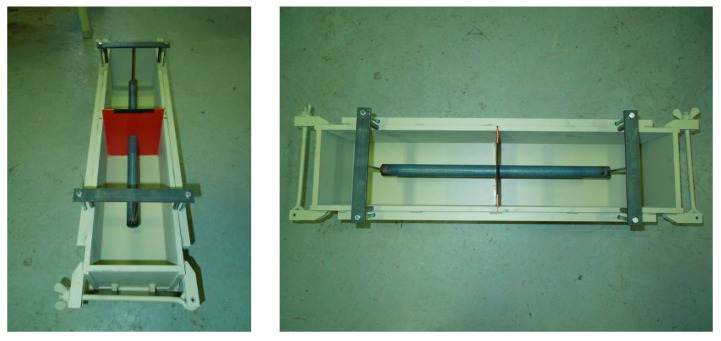
Installation of dowel in steel molds with a plastic partition in the middle and a device for dowel fixation in accurate position before casting for beams “A” (for dimensions of beams 150 × 150 × 350 mm).

A concrete mixture was prepared in laboratory. The concrete used in this test was prepared in accordance with the design of mixtures for constructions of highways by the Road and Motorway Directorate of the Czech Republic. The selected water to cement ratio was 0.40, and the used cement was CEM I 42.5 R, which was manufactured by Heidelberg Cement Group. The coarse aggregate was granite, gradation category Gc 90/15, with nominal maximum aggregate size of 22 mm. Fine aggregate was river sand complying with ČSN EN 933–1 and ČSN 736123–1 specification [20,25]. The superplasticizer additive, as an admixture, was used to improve installation and compacting of the concrete mixture. The fresh concrete parameters were set according to “ČSN EN 12350–2, Testing fresh concrete—Part 2: Slump test” and air content was set according to “ČSN EN 12350–7, Testing fresh concrete – Part 7: Air content”. Pressure methods were monitored during the testing [26,27]. After preparing the concrete, it was cast in molds without surface texture on the fresh concrete beams (Figure 1). The specimens were demolded after one day and were cured for 28 days at 20 °C and 100% relative humidity, and hardened concrete strength was monitored on cube and cylinder specimens according to “ČSN EN 12390 Testing hardened concrete—Part 3 and Part 6: Compressive/tensile splitting strength of test specimens” [28,29] and “ČSN 73 1318: Determination of tensile strength of concrete” [30], density parameters according to “ČSN EN 12390–7, Testing hardened concrete—Part 7: Density of hardened concrete”, and modulus of elasticity according to “ČSN ISO 1920–10: Testing of concrete—Part 10: Determination of static modulus of elasticity in compression” [31,32]. Table 1 shows mixture compositions and results of fresh and hardened concrete.

The specified expanded measurement uncertainty is a product of standard measurement uncertainty and the coverage factor k = 2, which corresponds with the coverage probability of approximately 95% for normal distribution. The standard measurement uncertainty was determined in compliance with the document EA 4/16 [33].

#### 2.1.2. Beams with Incorrectly Inserted Dowels “A”

In order to verify theoretical modelling results of tensile stress of concrete around dowels, static and cyclic tests were performed on concrete beams with dimensions of 150 × 150 × 350 mm with selected cases of defective positions of dowels:

- basic position (in the middle of the beam);
- vertical translation (downwards—20 mm);
- vertical tilt (towards the force—20 mm).

The aim of the tests was to find whether the dowel position in the slab has an impact on tensile stress in the vicinity of a dowel (particularly the existence of high tensile stress) and whether there is a risk of concrete damage. Findings from American tests were extended and beams with defective positions of dowels were tested. The selected variants were those that showed the highest stress in concrete by the theoretical calculation of the finite element method by ANSYS software and whose built-in dowel position allowed the installation of strain gauges [14,34].

**Figure 2:**
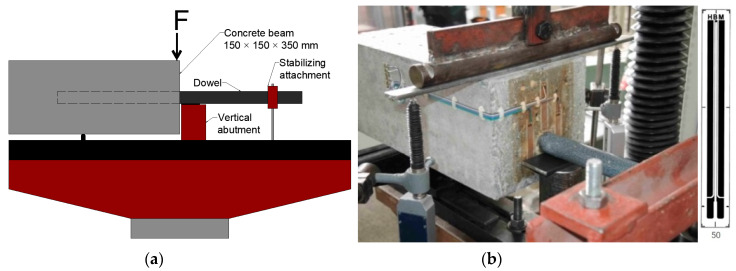
(**a**) Czech loading scheme and (**b**) loading test of beam “A” with 50 mm strain gauges (vertical translation).

The scheme of the loading test is based on an American study and the tests performed at West Virginia University, USA. The study confirmed occurrence of high concentrations of stress in concrete in the vicinity of dowels located in the central line plane of the concrete beam. The loading scheme for tests created by CDV’s researchers is shown in Figure 2a and represents a common situation with an insufficient support of the base on the transversal joints. The concrete beam and dowel are simply supported by vertical abutment. The stabilizing mechanism of the dowel allows free displacement and is used for safety only during laboratory testing [13].

The measured relative concrete deformations were determined with the use of glued strain gauges. The results were converted to stress according to Hooke’s Law and compared. Tensile and compressive stress σ is considered linearly proportional to its fractional extension or strain ε by the modulus of elasticity E:

(1) σ=Eε

where σ is the stress and E is the modulus of elasticity obtained from concrete tests.

Adhesive strain gauges were used for these tests. They are applied onto clean, dry and flat base of the concrete beam face in areas with the highest stress concentration, theoretically determined by the finite element method [34] 50 and 20 mm strain gauges were installed on the beam faces in the tensile (left and ride side) and the (above dowel) compression-stressed area (Figure 2b).

The principle of the static test was gradual loading of the beam by force applied by the head of the press machine and located above the dowel. The load increment was selected to be 50 N/s, the deflection of the top of concrete beam were monitored by displacement sensors and values of measured relative deformations by strain gauges. This procedure allowed to accurately determine relative deformations and deflections in relation to the applied force.

#### 2.1.3. Beams with Base Position of Dowel “B”

**Figure 3:**
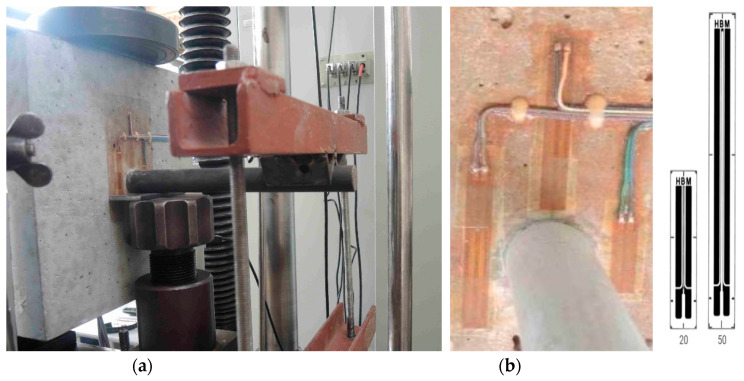
(**a**) Loading test of beam “B” and (**b**) applied strain gauges of 50 and 20 mm.

Beams “B”, so-called “superbeams” with dimensions of 270 × 270 × 300 mm were already tested with the aim of simulating loading in the scale 1:1 to the real road pavement thickness (Figure 3). After assessing dowels on beams “A”, a significant compression of the concrete edge into the epoxy coating was found (see Section 2.1.4). Therefore, dowels with hard coating were produced on the basis of fiberglass, smooth steel and more resistant thermoplastic coating (TUR), which were tested simultaneously with the original dowels. Results in five testing variants were compared and the effect of the type of coating was analyzed. Results of concrete stress around dowels and simulated behavior in joints provided us with a comparison and recommendation for the use of coatings in future. The boundary test conditions were identical to those of beam “A” (force, load increment, loading frequency, etc.).

#### 2.1.4. Coating Resistance

**Figure 4:**
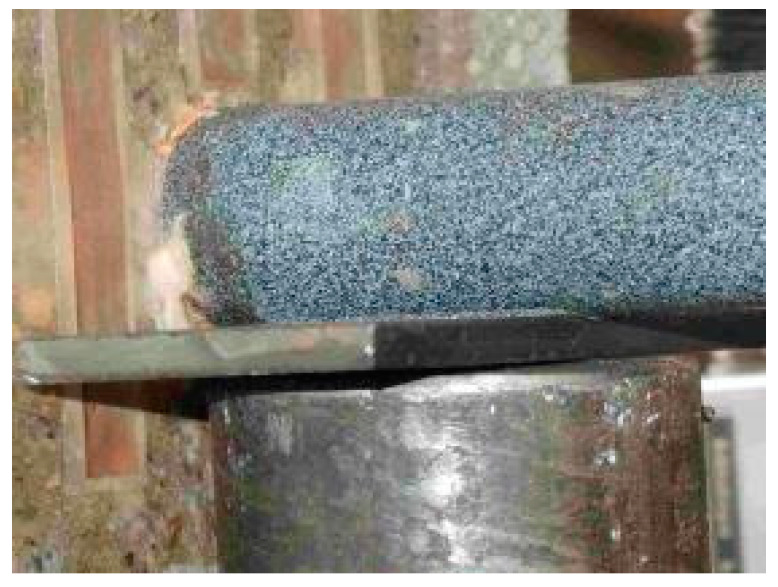
Extrusion of polyethylene (PE) plastic dowel coating during loading test of beam with inserted dowel.

Coating of dowels used nowadays in constructions of highway pavements and airports is produced from thermoplastic powder on the basis of modified polyolefins (marked PE plastic), as mentioned above. Dowels with these coatings have been used in the Czech Republic since the beginning of cement concrete pavement construction in 1996. Therefore, laboratory tests also focused on the quality of this epoxy coating. A detailed analysis of possible causes for low values of interaction found the potential main cause to be the low hardness of dowel coatings, which may cause its squeeze and damage at the interface with the cement concrete. Low resistance of the existing coating was clearly proven by the following loading tests of beams with an inserted dowel (Figure 4). Considering these findings, the authors focused on development of new materials that would contribute to the application in practice (hard plastic TUR and fiberglass coating). Material properties were investigated by Shore (durometer) hardness test.

#### 2.1.5. Longitudinal Displacement of Dowels in Cement Concrete 

Adjacent concrete slabs expand and shrink due to temperature loading. Dowels are required to allow horizontal displacement of concrete slabs. The aim of the tests was to assess the longitudinal displacement of dowels in the cement concrete.

**Figure 5:**
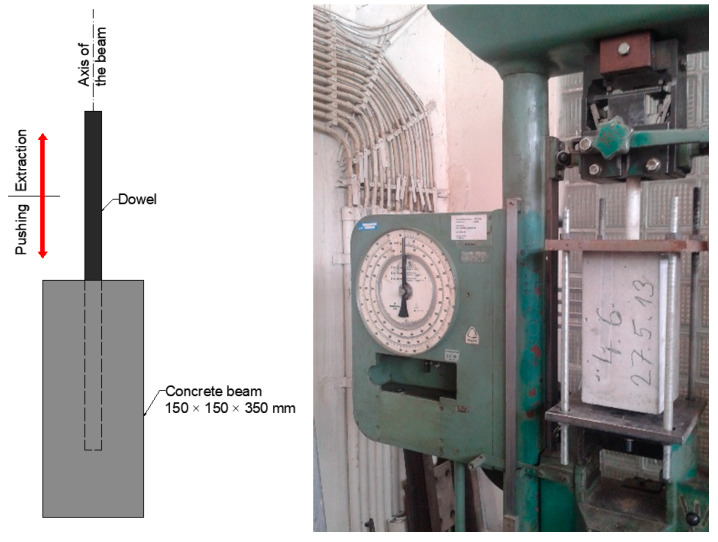
Longitudinal displacement—test of extracting/pushing of dowels into the concrete beam.

The tests were performed by gradual extracting and pushing of the dowel in a modified tensile testing machine. This test is not a standardised method and is used for a relative comparison of the force necessary to pull out and push back dowels to the beam under different temperatures and use of different dowel types. The maximum of ten cycles of gradual extracting and pushing of dowels into the beam were performed (Figure 5).

### 2.2. Slovak Loading Test

Regarding solutions to theoretical issues in the field of concrete road pavement behavior and reinforcement of transversal joints, an experiment in cooperation with Slovakia and Poland was performed with an aim to determine concrete slab behavior in relation to different designed diameters of dowels. The experiment was applied to currently ongoing construction of highway S8 Wroclaw–Warsaw.

**Figure 6:**
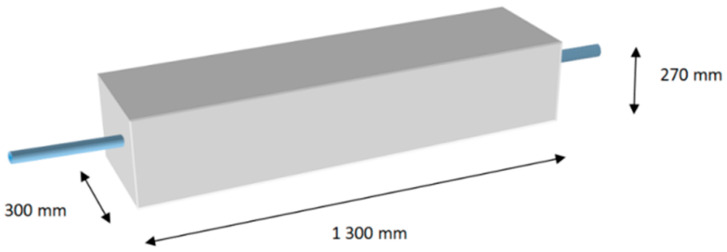
Testing beam.

Six testing specimens were designed and produced directly in the construction site of the highway for testing. Their dimensions were as follows (Figure 6): beam height was 270 mm (identical to the concrete slab thickness), beam width was 300 mm, which corresponded with the dowel spacing distance, and beam length was 1300 mm.

Concrete B40 (C35/40) was used for the beam production. The concrete was produced directly in the concrete plant in the construction site with the original recipe that was designed and used for highway S8 Wroclaw–Warsaw. In the experiment, the used dowels were those with the PE coating. They were produced from smooth steel S 235 JR with the diameters of 20, 25, 30, 40 mm and length of 500 mm, PE coating thickness of 0.3 mm in compliance with standard EN 13877-3.

The experiment focused on properties and behavior of a steel dowel in a concrete beam in terms of overall durability, deformation properties and potential beam damage type. Forces applied to a single dowel were determined according to an analytical model derived from the solution by Timoshenko and Lessels, for a differential equation of the deflection of a beam on an elastic foundation [35].

**Figure 7:**
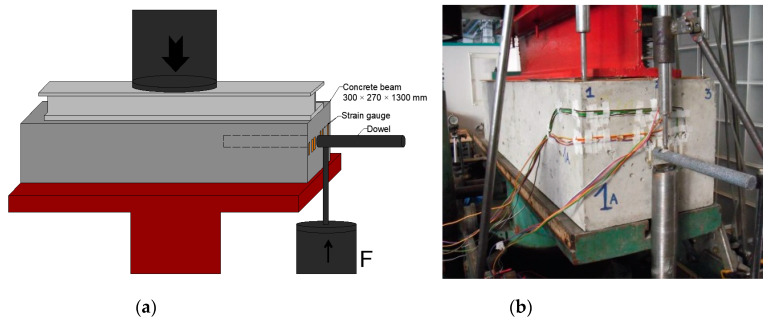
Slovak testing: (**a**) static loading test scheme and (**b**) real loading test.

A loading system (Figure 7a) was prepared for the needs of this experiment. The system consisted of a loading press and a loading mechanism used for producing the required force that represented a force effect on a single dowel. The principle of the static loading test was gradual loading of the beam by force located under the dowel (Figure 7b). Strain gauges 30 mm-long were installed on the “beam face”.

## 3. Results and Discussion

### 3.1. Czech Loading Test Results

#### 3.1.1. Results on Beam “A”

Table 2 shows a comprehensive overview of measurement results of static loading deformations of smaller beams with incorrect dowel positions, when the force increment was chosen to be 50 N/s. Deflections of the loaded beam edge were measured by digital deflectometers. Deflections of the unloaded edge were not measured as it was firmly supported. The tensile stress results show the value for 50 mm-long strain gauges (left of the dowel) and 20 mm-long strain gauges (right of the dowel) in brackets. The compressive stress results show the value for a 50 mm-long strain gauge (above the dowel).

The results in Table 2 make it clear that dowel positions have a substantial impact on damage in concrete. In the case of a dowel with vertical tilt (towards the force), tensile stress grows up to more than 100% against the base position (force 10 kN). Vertical translation of dowel has no significant impact on tensile stress increase but may negatively affect the load transfer efficiency of adjacent concrete slabs as well as further misalignment (horizontal skew, horizontal/longitudinal translation). Compressive stress on beams does not reach high values and there will be no concrete failure. Loaded edge deflection differences are negligible and comparable with measurement deflection on roads by falling weight deflectometers [34]. There was no significant displacement of the beam during the measurement.

#### 3.1.2. Results on Beam “B”

Table 3 shows a comprehensive overview of the deformation measurement results from static loading of super beams, when the force increment was chosen to be 50 N/s. Deflections of the loaded beam edge were measured by digital deflectometers. Deflections of the unloaded edge were not measured as the edge was firmly supported. The tensile stress results show the value for 50 mm-long strain gauges (left of the dowel) and 20 mm-long strain gauges (right of the dowel) in brackets. The compressive stress results show the value for a 50 mm-long strain gauge (above the dowel).

The results in Table 3 make it clear that dowel coating types have a substantial impact on stress in concrete. A dowel with coating from fiberglass, or a dowel without coating, show lower values of tensile and compressive stresses. In the case of dowels with PE plastic coating, tensile stress increases by more than 40–50% when compared with Fiberglass and TUR (force 10 kN). Compressive stress on beams does not reach high values and there will be no concrete failure, as was the case for beams “A”. Loaded edge deflection differences are negligible and comparable with measurement deflection on roads by falling weight deflectometers [34]. There was no significant displacement of the beam during the measurement, except for No. 5, where the dowel coating was compressed during the application of loading. This phenomenon is described in Section 2.1.4, Figure 5.

#### 3.1.3. Longitudinal Displacement of Dowels in Cement Concrete 

Dowel coatings with PE plastic, new plastic TUR, and new fiberglass with special surface treatment that should ensure dowel longitudinal displacement in the beam were tested. The comparison of results in Table 4 shows that the new TUR coating has lower resistance during longitudinal displacement of the dowel in concrete (from the maximum value of 22 kN at extraction, the force decreases to 10.8 kN), therefore, it is more suitable than the existing coating, which has so far been suitable and hindered no expansion movements of concrete slabs due to changes in average temperatures of the concrete. The extraction force for the currently used dowel with the epoxy coating was determined to be 60–70 kN, and this force was gradually decreasing.

**Figure 8:**
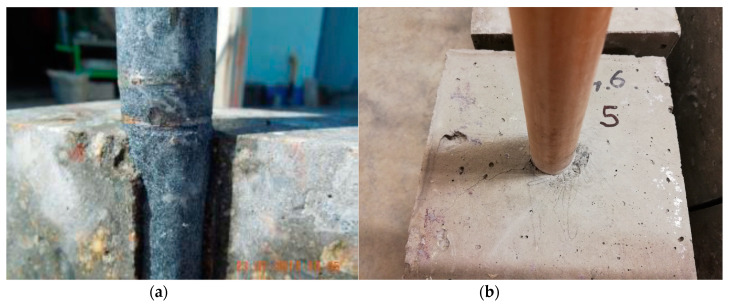
Beam after longitudinal displacement test: (**a**) damage of the PE plastic coating; (**b**) damage of concrete after testing (fiberglass dowel).

Table 4 also implies that the PE plastic coating has approximately the same resistance during the longitudinal displacement of the dowel in concrete as fiberglass. Therefore, it means that the TUR coating has better properties than the existing coating in this respect than the fiberglass coating. Moreover, analyses of coatings after the laboratory testing show the damage of the PE plastic coating to the dowel (Figure 8a) and also beams with the fiberglass dowel (Figure 8b) after laboratory testing.

### 3.2. Slovak Loading Test Results

**Figure 9:**
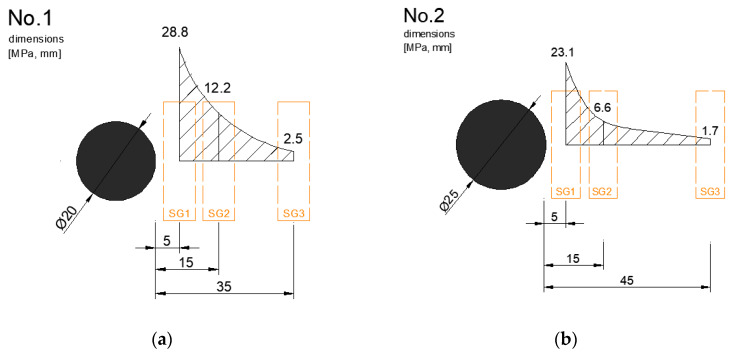
Stress results on testing beams (force 10 kN): (**a**) No.1—dowel Ø 20 mm; (**b**) No.2—dowel Ø 25 mm.

**Figure 10:**
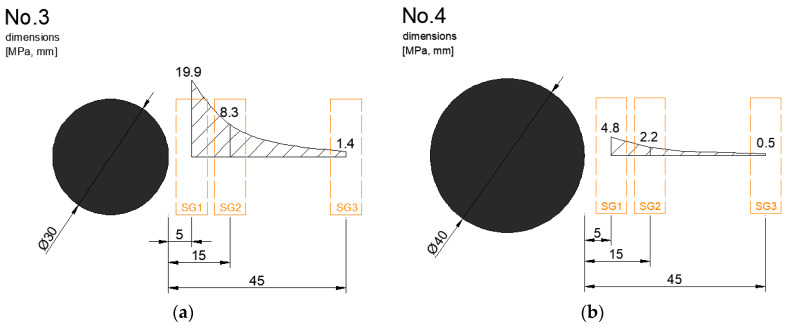
Stress results on testing beams (force 10 kN): (**a**) No.3—dowel Ø 30 mm; (**b**) No.4—dowel Ø 40 mm.

Static loading test results for individual beams are shown in more detail in Table 5. Normal stress σ at testing beams and at steel dowels was evaluated through measurements of relative deformations ε. The position of strain gauges (SG) were chosen taking into account the possibilities of each beam. The beams were installed in the construction site and demolded in laboratory. The positions of strain gauges observe deformations of the concrete slab “face” (Figure 9 and Figure 10).

**Figure 11:**
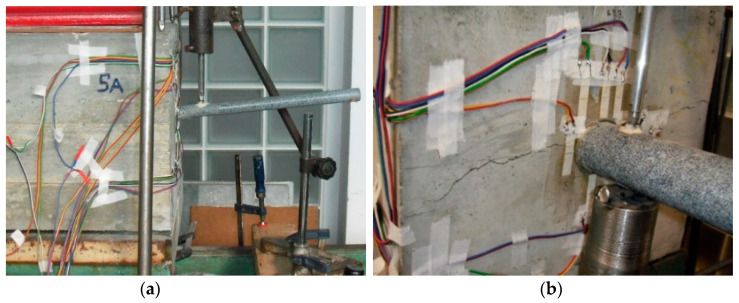
Damage of testing beam: (**a**) No.1—dowel Ø 20 mm; (**b**) No.4—dowel Ø 40 mm.

During gradual loading up to the state when the beam with the lowest diameter (Ø 20 mm) was damaged, its deformation occurred already at the force of 10.3 kN and loading could not continue. The concrete beam remained undamaged (Figure 11a). In the case of a dowel with diameter Ø 40 mm, the maximum reached force of 50 kN resulted in a horizontal crack through the concrete beam around the dowel up to the distance of 250 mm—cast-in length of the dowel (Figure 11b).

## 4. Conclusions and Discussions

This paper presents the main findings of laboratory testing of dowels used in the Czech Republic and Slovakia. The results show high tensile stress in concrete around dowels and confirm the results of theoretical modelling by FEM. Laboratory test results showed high stress around the dowel, which is higher for the traditional diameter of 25 mm than for the diameters of 30 and 40mm. Tensile stress values were experimentally determined in concrete for loading of 10 kN, which is the maximum force that traffic loading may be applied to a dowel. Since the tensile strength of concrete is lower (Table 1) than the measured values of tensile stress, concrete damage may occur in the area of the transversal joint particularly for 25 mm dowels [34]. It is recommended to increase the dowel diameter for JPCPs on highways (thickness 250–300 mm) to 30 mm while keeping the length of 500 mm and spacing of 250 mm in slow lanes, which are the most heavily loaded. The spacing may be extended to 500 mm in fast lanes. The findings were integrated into TP 098: Design of concrete pavements on motorways in Slovakia. 

The effects of defective positions of dowels in critical variants were analyzed. The downward vertical translation of the dowels by 20 mm has no major effect on tensile stress results. The highest tensile stress values were examined in the variant with the vertical tilt (20 mm) of the dowel. Therefore, it is essential to maintain the specified position with tolerances given in standard ČSN 736123-1, with particular attention paid to the vertical and horizontal tilt of the dowel. The number of defectively installed dowels in every transversal joint must be taken into account and their effect on road pavement durability must be assessed, e.g., by pavement modelling with the use of the finite element method.

New coating types with higher hardness and resistance to mechanical damage were tested. Higher quality of monitored parameters, such as longitudinal displacement of dowels in concrete and resistance against loading, was reached and verified with the use of newly developed nonstandard testing. Fiberglass based coatings failed to prove to be effective in terms of longitudinal displacement in concrete and they would probably work in the structure as anchors. Therefore, damage may occur during the JPCP expansion. A new type of coating of dowels, TUR, has lower values of tensile stress around the dowels and is currently fully used in the constructions and repairs of JPCPs on highways and in airports.

## Figures and Tables

**Table 1:** Material parameters of concrete.

**Component**	**Batch Proportions (kg/m^3^)**	**Locality**
CEM I 42.5 R	375	Mokra
Aggregate 0/4	712	Tovacov
Aggregate 4/8	178	Olbramovice
Aggregate 8/16	534	Olbramovice
Aggregate 11/22	356	Olbramovice
Superplasticizer	3	-
Water	150	-
**Fresh concrete Testing**	**Value (uncertainty)**	**-**
Slump test	30 mm (±10 mm)	-
Air content	5.9% (±0.2%)	-
**Hardened Concrete testing**	**Value (uncertainty)**	**-**
Compressive strength	70.4 MPa (±1.9 MPa) *	-
Split tensile strength	5.6 MPa (±0.4 MPa) *	-
Direct tensile strength	2.9 MPa (±0.6 MPa) *	-
Density	2372 kg/m^3^ (±30 kg/m^3^) *	-
Modulus of elasticity	35,900 MPa (±600 MPa) *	
w/c ratio	0.40 (-)	-

* Average of three specimens.

**Table 2:** Deformation measurement results on beams 150 × 150 × 350 mm.

No.	1	2	3
Coating	PE Plastic		
Diameter (Type, mm)	Smooth Steel, 25		
Dowel Position	in the Middle	Vertical Translation (Downwards—20 mm)	Vertical Tilt (towards the Force—20 mm)
**Producer**	Czech Republic		
**Force [kN]**	Tensile stress [MPa] on strain gauges of 50 (20) mm		
2	0.01 (0.01)	0.02 (0.01)	0.16 (0.19)
4	0.16 (0.26)	0.17 (0.19)	0.52 (0.66)
6	0.38 (0.62)	0.43 (0.47)	0.94 (1.19)
8	0.62 (1.01)	0.72 (0.83)	1.35 (1.73)
10	0.91 (1.42)	1.03 (1.23)	1.87 (2.33)
**Force [kN]**	Compressive stress [MPa] on strain gauge of 50 mm		
2	2.99	1.27	2.15
4	6.41	2.94	4.90
6	9.76	4.70	7.55
8	13.04	6.58	10.23
10	16.24	8.59	12.95
**Force [kN]**	Loaded edge deflection [mm]		
2	0.186	0.180	0.120
4	0.202	0.232	0.208
6	0.302	0.280	0.288
8	0.392	0.334	0.358
10	0.442	0.386	0.408

**Table 3:** Deformation measurement results on beams 270 × 270 × 300 mm.

No.	1	2	3	4	5
Coating	–	PE Plastic	Fiberglass	TUR	PE Plastic
Shore A Hardness Test (-)	-	50	90	75	50
Diameter (Type, mm)	Smooth Steel 25				
Dowel Position	in the Middle of Beam				
**Producer**	Czech Republic	Czech Republic	Czech Republic	Czech Republic	Germany
**Force [kN]**	Tensile stress [MPa] on strain gauges of 50 (20) mm				
2	0.15 (0.25)	0.15 (0.35)	0.03 (0.10)	0.16 (0.20)	0.26 (0.24)
4	0.36 (0.73)	0.50 (1.19)	0.20 (0.59)	0.45 (0.66)	0.70 (0.94)
6	0.62 (1.31)	0.88 (2.17)	0.38 (1.17)	0.76 (1.14)	1.17 (1.76)
8	0.90 (2.19)	1.26 (3.19)	0.63 (1.96)	1.13 (1.71)	1.70 (2.69)
10	1.23 (3.32)	1.65 (4.22)	0.91 (2.78)	1.55 (2.37)	2.30 (3.93)
**Force [kN]**	Compressive stress [MPa] on strain gauge of 50 mm				
2	1.20	1.11	1.01	0.93	0.71
4	2.51	3.12	2.35	2.40	2.31
6	3.85	5.11	3.69	3.90	4.02
8	5.31	7.04	5.16	5.53	5.73
10	6.82	8.95	6.62	7.28	7.73
**Force [kN]**	Loaded edge deflection [mm]				
2	0.072	0.102	0.036	0.030	0.136
4	0.114	0.154	0.106	0.088	0.210
6	0.150	0.204	0.154	0.128	0.274
8	0.176	0.250	0.198	0.170	0.328
10	0.204	0.288	0.240	0.210	0.366

**Table 4:** Longitudinal displacement results on beams 150 × 150 × 350 mm.

Type	Test Number	Dowel Diameter [mm]	Maximum Extraction Force [kN]	Maximum Extrusion Force [kN]
PE Plastic	1	25	64.0	42.0
	2		37.8	34.0
	3		32.6	29.6
	4		29.2	25.8
	5		26.4	22.8
	6		24.7	20.5
	7		22.0	18.3
	8		20.8	16.8
	9		18.8	15.3
	10		17.4	13.5
TUR	1	25	22.0	17.0
	2		14.6	14.0
	3		13.2	14.0
	4		12.7	14.0
	5		12.5	13.6
	6		12.5	13.2
	7		12.0	12.8
	8		11.4	12.4
	9		11.2	12.4
	10		10.8	12.4
Fiberglass	1	25	62.7	69.1
	2		46.8	45.0
	3		44.3	41.2
	4		43.3	39.9
	5		42.5	38.3
	6		39.8	38.5
	7		39.1	34.3
	8		37.3	33.2
	9		28.3	33.7
	10		26.3	32.3

**Table 5:** Stress distribution at faces of concrete beams, force 10 kN.

No.	Dowel Diameter (mm)	Stress (MPa)		
		SG1	SG2	SG3
1	20	28.8	12.2	2.5
2	25	23.1	6.6	1.7
3	30	19.9	8.3	1.4
4	40	4.8	2.2	0.5

## References

[1] AASHTO (1993). Guide for Design of Pavement Structures.

[2] Zienkiewicz O., Taylor R. (1994). The Finite Element Method, Fourth Edition; Volume 1: Basic Formulation and Linear Problems.

[3] Mackiewicz P. (2015). Analysis of stresses in concrete pavement under a dowel according to its diameter and load transfer efficiency. Can. J. Transp. Eng..

[4] Huang Y.H. (1974). Finite element analysis of slabs on elastic solids. J. Transp. Eng..

[5] Tabatabai A.M., Barenberg E.J. (1978). Finite-Element Analysis of Jointed or Cracked Concrete Pavements.

[6] Tayabji S.P., Colley B.E. (1986). Analysis of Jointed Concrete Pavements.

[7] Shoukry S.N., Fahmy M., Prucz J., William G. (2007). Validation of 3DFE analysis of rigid pavement dynamic response to moving traffic and nonlinear temperature gradient effects. Int. J. Geomech..

[8] Rousseau J., Frangin E., Marin P., Daudeville L. (2009). Multidomain finite and discrete elements method for impact analysis of a concrete structure. Eng. Struct..

[9] Ding F., Yin Y., Cai L., Tang Y., Wang X. (2017). Mechanical Response of Typical Cement Concrete Pavements under Impact Loading. Math. Probl. Eng..

[10] Sadeghi V., Hesami S. (2017). Investigation of load transfer efficiency in jointed plain concrete pavements (JPCP) using FEM. Int. J. Pavement Res. Technol..

[11] Li G., Chen C., Jiang B., Shen Q. (2014). Development of prediction model for doweled joint concrete pavement using three-dimensional finite element analysis. Appl. Mech. Mater..

[12] Mokhatar S., Abdullah R., Kueh A. (2013). Computational impact responses of reinforced concrete slabs. Comput. Concr..

[13] Khazanovich L., Neeraj B., Alex G. (2001). Evaluation of Alignment Tolerances for Dowels and Their Effects on Joint Performance.

[14] Shoukry S.N., William G.W., Riad M. Application of LS-DYNA in Identifying Critical Stresses Around Dowels. Proceedings of the 8th International LS-DYNA Users Conference.

[15] Li L., Tan Y., Gong X., Li Y. Characterization of Contact Stresses between Dowels and Surrounding Concrete in Jointed Concrete Pavement. Proceedings of the 10th International Conference on Concrete Pavements.

[16] NCC (2011). Recommendations for Standardization of Dowel Load Transfer Systems for Jointed Concrete Roadway Pavements.

[17] NCHRP (2009). Report 637: Guidelines for Dowel Alignment in Concrete Pavements.

[18] Larson R., Smith K. (2011). Evaluation of Alternative Dowel Bar Materials and Coatings.

[19] TP 098 (2015). Design of Concrete Pavements on Motorways.

[20] ČSN 736123–1 (2014). Road Building—Concrete Pavements—Part 1: Construction and Conformity Assessment.

[21] EN 13877–1 (2006). Concrete Pavements—Part 1: Materials.

[22] EN 13877–3 (2006). Concrete Pavements—Part 3: Specification for Dowels to Be Used in Concrete Pavements.

[23] EN ISO 15630–v1 (2020). Steel for the Reinforcement and Prestressing of Concrete–Test Methods–Part 1: Reinforcing Bars, Rods and Wire.

[24] EN 10060 (2004). Hot Rolled Round Steel Bars for General Purposes–Dimensions and Tolerances on Shape and Dimensions.

[25] ČSN EN 933–1 (2012). Tests for Geometrical Properties of Aggregates–Part 1: Determination of Particle Size Distribution–Sieving Method.

[26] ČSN EN 12350–2 (2009). Testing Fresh Concrete–Part 2: Slump-Test.

[27] ČSN EN 12350–7 (2009). Testing Fresh Concrete–Part 7: Air Content–Pressure Methods.

[28] ČSN EN 12390–3 (2019). Testing Hardened Concrete–Part 3: Compressive Strength of Test Specimens.

[29] ČSN EN 12390–6 (2010). Testing Hardened Concrete–Part 6: Tensile Splitting Strength of Test Specimens.

[30] (1994). ČSN 73 1318: Determination of Tensile Strength of Concrete–Z1.

[31] (2019). ČSN EN 12390-7, Testing Hardened Concrete–Part 7: Density of Hardened Concrete.

[32] (2016). ČSN ISO 1920-10: Testing of Concrete–Part 10: Determination of Static Modulus of Elasticity in Compression.

[33] EA-4/16 (2003). Guidelines on the Expression of Uncertainty in Quantitative Testing.

[34] Grosek J., Zuzulova A., Brezina I. (2019). Effectiveness of Dowels in Concrete Pavement. Materials.

[35] FHWA-HRT-06-106 (2009). Design and Evaluation of Jointed Plain Concrete Pavement with Fiber Reinforced Polymer Dowels.

